# The effect of missing teeth on dementia in older people: a nationwide population-based cohort study in South Korea

**DOI:** 10.1186/s12903-019-0750-4

**Published:** 2019-04-25

**Authors:** Jin-Joo Yoo, Joon-Ho Yoon, Min-Jin Kang, Manyong Kim, Namsik Oh

**Affiliations:** 1 Department of Prosthodontics, National Health Insurance Service Ilsan Hospital, 100 Ilsan-ro, Ilsandong-gu, Goyang-si, Gyeonggi-do 10444 Republic of Korea; 2 Department of Policy Research Affairs, National Health Insurance Service Ilsan Hospital, 100 Ilsan-ro, Ilsandong-gu, Goyang-si, Gyeonggi-do 10444 Republic of Korea; 3 Department of Dentistry, College of Medicine, Inha University, 27, Inhang-ro, Jung-gu, Incheon, 22332 Republic of Korea

**Keywords:** Dementia, Tooth extraction, Tooth loss, Cohort studies, Periodontal diseases

## Abstract

**Background:**

To determine the effect of missing teeth on the risk of dementia onset among individuals who received tooth extractions and those who did not, based on the number of missing teeth.

**Methods:**

We selected individuals who had not been diagnosed or treated for dementia between 2002 to 2011 from the National Health Insurance Service-Elderly Cohort Database (NHIS-ECD). We divided participants into two cohorts, a tooth extraction and non-extraction cohort, based on tooth loss from 2002 to 2011. After propensity score matching, there were 104,903 individuals in each cohort, and we included a total of 209,806 individuals in this study. Each cohort was grouped by sex, age, residential area, health insurance eligibility, income level, history of dental caries, history of periodontal treatment, and number of extracted teeth. We analyzed the relationship between dementia onset and these variables using logistic regression analysis.

**Results:**

Individuals with tooth loss had a higher risk for dementia than those without tooth loss (odds ratio [OR] = 1.18; 95% confidence interval [CI]: 1.146–1.215). Regarding the incidence of dementia, the OR increased as the number of missing teeth and age increased, and the OR was higher for women (OR = 1.33; 95% CI: 1.286–1.367) than for men, and this difference was statistically significant (*P* < 0.01). The incidence of dementia decreased with periodontal treatment (OR = 0.96; 95% CI: 0.932–0.992) and increased with dental caries (OR = 1.07; 95% CI: 1.035–1.101).

**Conclusions:**

These results suggest that it is important to delay tooth loss and preserve the stable remaining teeth to help prevent dementia.

**Electronic supplementary material:**

The online version of this article (10.1186/s12903-019-0750-4) contains supplementary material, which is available to authorized users.

## Background

By 2015, approximately 46.8 million people worldwide were reported to have dementia, costing an estimated 818 billion USD in 2015, roughly equivalent to 1% of the global gross domestic product [1]. It is estimated that about 4.6 million new cases of dementia occur yearly. According to this projection, the number of affected patients will triple to about 131.5 million by 2050 [1]. The World Health Organization has reported that dementia profoundly affects the patient’s family or caregiver, causing excess stress from physical, mental, and economic pressures [2]. As of 2016, there were 690,000 patients with dementia in Korea, and an estimated 2.7 million family members, including spouses, children, and grandchildren, who cared for them. As of 2016 in Korea, 57 people capable of producing income (i.e., producing population) were available to care for a patient with dementia, but this is expected to decrease to 7.4 people per dementia patient by 2060, due to a declining producing population within the aging population [3]. The social burden of patients in Korea is expected to increase substantially and will also likely be felt globally.

Previous studies have linked tooth loss with dementia [4–19]. It was reported that cognitive function significantly decreased as the number of remaining teeth decreased [4, 13, 14, 16]. If teeth are lost, chewing efficiency and function are reduced, which decreases the ability to perform everyday activities and the quality of life and increases the risk of conditions like depression [20–22]. Also, the reduction of chewing functions might decrease learning and memory abilities [23–25].

Health insurance coverage is provided for all residents of Korea because social insurance and medical benefits are provided as public assistance, and exceptions are specified in the National Health Insurance Act [26]. The NHIS has maintained national records of medical use and prescriptions. Based on this, the NHIS–National Sample Cohort (NHIS-NSC) database has recently been established in South Korea and actively used to conduct studies [27]. The NHIS-NSC database includes medical information about 1 million people, or 2.2% of the whole South Korean population. In addition, there are established databases for a medical check-up cohort, a working woman cohort, an infant medical check-up cohort, and an elderly cohort with different subjects and information, depending on the purpose. These population databases facilitate cohort studies because they allow disease groups and control groups to be set up relatively easily, and they increase the reliability of research results with large sample sizes.

As social interest in dementia has increased, dental researchers have studied the relationship between dementia and oral diseases [4–15, 28–32]. However, these studies had limited sample sizes, and any large-scale epidemiological relationship is still unrecognized. Therefore, this study aimed to analyze the relationship between dementia and periodontal disease, dental caries, and tooth loss using the elderly cohort database. The null hypothesis is that the risk of dementia is the same regardless of the history of tooth extraction.

## Methods

### Study design and data collection

In this population-based cohort study, we analyzed the risk factors of dementia in older people over 60 years old, using the Elderly Cohort Database (ECD) of the NHIS. This resource supports studies involving older adults, such as investigations of risk factors and prognosis of geriatric diseases. The ECD contains 558,147 individuals, which is 10% of the older people over 60 years of age who had health insurance and medical benefits at the end of 2002 [33]. It covers information for 14 years, from 2002 to 2015, including socio-economic qualification variables, medical resource utilization and type of clinic. Data on long-term nursing services for older adults was recorded from 2008 to 2015.

### Research subjects

**Fig. 1:**
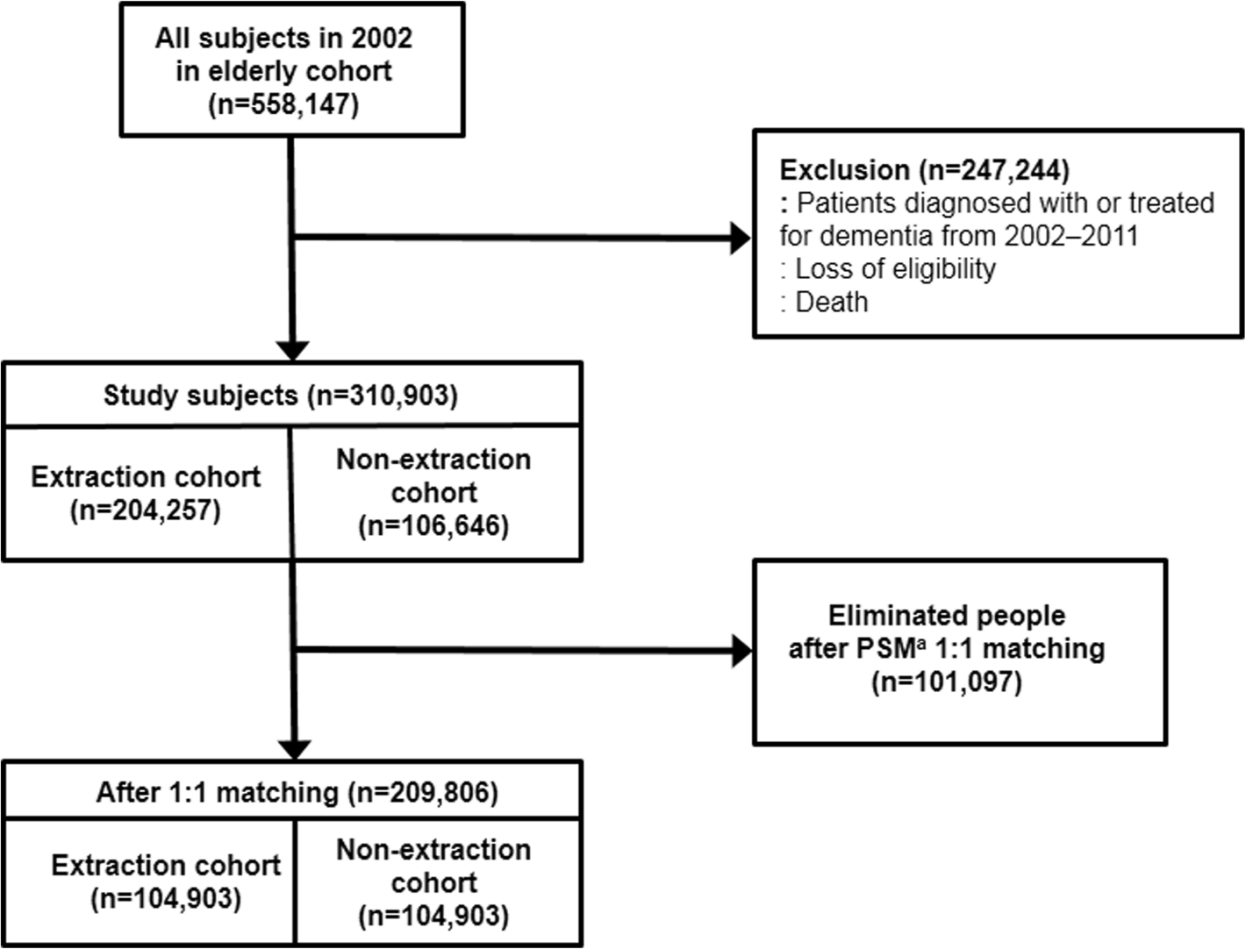
Flow chart for selection of subjects. ^a^Propensity score matching

Of the 558,147 subjects in the ECD in 2002, we excluded those who had been diagnosed or treated for dementia in 2002–2011, had died, or were disqualified within the study period. We divided the remaining subjects into two groups, the extraction cohort and the non-extraction cohort, based on the number of tooth loss from 2002 to 2011. The extraction cohort included those with a history of tooth extraction, according to the treatment database of the ECD. The non-extraction cohort included those without a history of extraction during the same period. As a result, there were 204,257 subjects in the extraction cohort and 106,646 subjects in the non-extraction cohort. To compare the extraction cohort and the non-extraction cohort, we used propensity score matching to match non-extraction individuals with extraction individuals 1:1 (Fig. 1). After matching, there were 104,903 individuals in each cohort and a total of 209,806 individuals in the study (Fig. 1). We defined individuals with dementia as having a dementia diagnosis or more than one dementia-related treatment. To limit our sample to individuals diagnosed with dementia for the first time during the study period, we excluded those who were diagnosed with dementia from January 2002 through December 2011 and included only those who visited the hospital with dementia for the first time during January 2012 through December 2013. Non-demented patients were defined as those without a history of dementia diagnosis or treatment during the entire observation period.

### Variables

In this study, exposure variables were a history of tooth extraction and the number of extracted teeth and the outcome variable was the onset of dementia from January 2012 through December 2013. Age, gender, socio-economic factors, and history of dental caries or periodontal disease were considered as confounding variables. Socio-economic factors included income, residential area, and insurance eligibility.

Age was sorted into three groups: 60–69, 70–79, and ≥ 80 years old. Income was divided into five groups based on the income quintile. That is, the first quintile is the bottom 20% of income, and the fifth quintile is the top 20% of income. For residential area, we classified residents of metropolitan as urban residents, whereas those living in other areas were classified as rural residents. We classified the insurance eligibility of the subjects in each cohort as either the head of the household, family member, or medical aid beneficiaries. We grouped extraction individuals into three groups depending on the number of teeth removed: individuals with 1–6 teeth removed, 7–12 teeth removed, and ≥ 13 teeth removed.

### Operational definition of diseases

Treatment codes and operational definitions associated with tooth extraction are listed in supplementary material (See Additional file 1. We used the following three treatment codes and the operational definition of extraction: U4412 (extraction-anterior tooth), U4413 (extraction-posterior tooth), and U4414 (extraction-complicated extraction).

For the operational definition of dementia, we referred to treatment details (30 t) and type of disease (40 t) in the treatment database. We included all types of dementia in this study (See Additional file 2).

Dental-related diseases were defined as follows. Patients with periodontal disease were defined as those who had been diagnosed with gingivitis and periodontitis (See Additional file 3) from 2002 to 2011, and who had a history of more than one periodontal treatment, as shown in supplementary material (See Additional file 4). Patients with dental caries were defined as those diagnosed with dental caries (See Additional file 5) and who went to a clinic at least once for treatment.

### Statistical analysis

We analyzed the data using SAS software (Version 9.4, SAS Institute Inc., Cary, NC) and used Chi-square tests to analyze descriptive statistics and the frequencies of various factors. We analyzed these factors using logistic regression analysis and performed detailed analysis by age and gender.

This study followed the Statement of Strengthening the Reporting of Observational Studies in Epidemiology (STROBE) guidelines (See Additional file 6) and was conducted after obtaining approval from the Institutional Review Board (IRB) of the NHIS Ilsan Hospital for general aspects of the research, including the collection and processing of research data and the study design. The IRB Approval number is: NHIMC 2017–01-034.

## Results

The demographic characteristics of each cohort, grouped according to whether they had teeth extracted or not, are shown in Table 1. The subjects included in this study ranged from 60 to 113 years, with an average age of 67.5 years. The average age was 68.5 for extraction cohort men and 66.5 for non-extraction cohort. We determined the number and percentage of subjects grouped by each variable when the tooth extraction cohort was divided into three groups by number of teeth removed (1–6 teeth, 7–12 teeth, and ≥ 13 teeth) during the 10 years in the study (Table 2). Regarding the incidence of dementia, the odds ratio (OR) was higher for women than for men, and the difference was statistically significant (*p* < 0.01). When grouped by age and using 60–69 years as the reference point, the OR for the 70–79 group was 1.88 (95% confidence interval [CI]: 1.827–1.938), and the OR for the ≥80 group was 2.38 (95% CI: 2.216–2.553); this difference was statistically significant (p < 0.01). The OR for urban residents (OR = 1.33; 95% CI: 1.294–1.367) was higher than for rural residents. The OR of medical aid beneficiaries was 1.50 (95% CI: 1.414–1.585) and was significantly different than the OR of the head of the household (*p* < 0.01). Regarding income, the second quintile was not significantly different than the first, lowest-income quintile, whereas the third, fourth, and fifth quintiles had a significantly lower OR than the first quintile (*p* < 0.05). This means that the percentages of individuals with dementia in the third, fourth, and fifth quintiles were lower than that in the first quintile. Those with a history of periodontal therapy had significantly lower ORs (OR = 0.96; 95% CI: 0.932–0.920; *p* = 0.01) for dementia incidence. By contrast, those with a history of dental caries had significantly higher ORs (OR = 1.07; 95% CI: 1.035–1.101; *p* < 0.01) for dementia (Table 3).

**Table 1:** Demographic characteristics of extraction and non-extraction cohorts

	Non-extraction Cohort		Extraction Cohort		*p*-value
	(people)	(%)	(people)	(%)	
Total	104,903		104,903		
Male	35,111	33.5	35,111	33.5	1.0
Female	69,792	66.5	69,792	66.5	
Age					1.0
60–69	76,570	73.0	76,570	73.0	
70–79	25,699	24.5	25,699	24.5	
≥ 80	2634	2.5	2634	2.5	
Residential Area					<.001^a^
Urban	59,487	56.7	59,487	56.7	
Rural	45,416	43.3	45,416	43.3	
Eligibility					<.001^a^
Head of household	44,292	42.2	43,901	41.8	
Family member	52,292	49.8	55,647	53.0	
Medical aid beneficiaries	8319	7.9	5355	5.1	
Income level					<.001^a^
First quintile	24,361	23.2	21,088	20.1	
Second quintile	13,851	13.2	12,681	12.1	
Third quintile	15,795	15.1	14,576	13.9	
Fourth quintile	21,811	20.8	22,506	21.5	
Fifth quintile	29,085	27.7	34,052	32.5	
History of dental caries					<.001^a^
No	74,615	71.1	50,473	48.1	
Yes	30,288	28.9	54,430	51.9	
History of periodontal treatment					<.001^a^
No	73,599	70.2	42,282	40.3	
Yes	31,304	29.8	62,621	59.7	
Onset of dementia					<.001^a^
No	93,711	89.3	92,051	87.7	
Yes	11,192	10.7	12,852	12.3	

^a^Statistically significant at a 95% confidence interval

**Table 2:** Population distribution according to the number of missing teeth in the tooth extraction cohort^a^

	The number of missing teeth						*p*-value
	1–6		7–12		≥13		
	(people)	(%)	(people)	(%)	(people)	(%)	
Total	84,411		17,341		3151		
Male	27,112	32.1	6613	38.1	1386	44.0	<.001^b^
Female	57,299	67.9	10,728	61.9	1765	56.0	
Age							
60–69	61,829	73.2	12,459	71.8	2282	72.4	<.001^b^
70–79	20,398	24.2	4492	25.9	809	25.7	
≥ 80	2184	2.6	390	2.2	60	1.9	
Residential area							
Urban	48,167	57.1	9589	55.3	1731	54.9	<.001^b^
Rural	36,244	42.9	7752	44.7	1420	45.1	
Eligibility							
Head of household	35,076	41.6	7426	42.8	1399	44.4	<.001^b^
Family member	44,844	53.1	9165	52.9	1638	52.0	
Medical aid beneficiaries	4491	5.3	750	4.3	114	3.6	
Income level							
First quintile	17,073	20.2	3386	19.5	629	20.0	<.001^b^
Second quintile	10,074	11.9	2201	12.7	406	12.9	
Third quintile	11,513	13.6	2561	14.8	502	15.9	
Fourth quintile	18,139	21.5	3729	21.5	638	20.2	
Fifth quintile	27,612	32.7	5464	31.5	976	31.0	
Experience of dental caries							
No	41,170	48.8	7898	45.5	1405	44.6	<.001^b^
Yes	43,241	51.2	9443	54.5	1746	55.4	
Experience of periodontal treatment							
No	35,306	41.8	5964	34.4	1012	32.1	<.001^b^
Yes	49,105	58.2	11,377	65.6	2139	67.9	
Onset of dementia							
No	74,230	87.9	15,083	87.0	2738	86.9	<.001^b^
Yes	10,181	12.1	2258	13.0	413	13.1	

^a^It is helpful to refer to Table 1 to see statistical differences from the sample population shown in this table

^b^Statistically significant at a 95% confidence interval

**Table 3:** Factors associated with the incidence of dementia

Effect		Odds Ratio Estimates			*p*-value
		Point Estimate	95% Wald Confidence Limits		
History of tooth extraction (standard = no history)		1.180	1.146	1.215	<.0001^a^
Female (standard = male)		1.326	1.286	1.367	<.0001^a^
Age	70–79 (standard = 60–69)	1.882	1.827	1.938	<.0001^a^
	≥80 (standard = 60–69)	2.378	2.216	2.553	<.0001^a^
Urban resident (standard = rural resident)		1.330	1.294	1.367	<.0001^a^
Eligibility	Family member (standard = head of household)	1.005	0.975	1.035	0.7616
	Medical aid beneficiaries (standard = head of household)	1.497	1.414	1.585	<.0001^a^
Income level	Second quintile (standard = first quintile)	0.980	0.930	1.032	0.4347
	Third quintile (standard = first quintile)	0.947	0.900	0.997	0.0368^a^
	Fourth quintile (standard = first quintile)	0.938	0.895	0.984	0.0080^a^
	Fifth quintile (standard = first quintile)	0.952	0.911	0.994	0.0266^a^
History of periodontal treatment (standard = no history)		0.962	0.932	0.992	0.0132^a^
History of dental caries (standard = no history)		1.067	1.035	1.101	<.0001^a^

^a^Statistically significant at 95% Confidence Interval

Table 4 shows the logistic regression analysis of the relationship between dementia incidence and the three groups divided according to the number of missing teeth. Compared with the control group with no missing teeth, the ORs of those with 1–6 teeth lost was 1.16 (95% CI: 1.124–1.294), those with 7–12 teeth lost was 1.27 (95% CI: 1.210–1.338), and those with ≥13 teeth lost was 1.31 (95% CI: 1.179–1.461), indicating that the OR for dementia significantly increased as the number of missing teeth increased (*p* < 0.01).

**Table 4:** Factors related to the incidence of dementia according to the number of teeth lost

Variables	Odds Ratio Estimates			*p*-value
	Point Estimate	95% Wald Confidence Limits		
The number of teeth lost				
1–6 (standard = 0)	1.158	1.124	1.194	<.0001^a^
7–12 (standard = 0)	1.272	1.210	1.338	<.0001^a^
≥13 (standard = 0)	1.313	1.179	1.461	<.0001^a^
Female (standard = male)	1.330	1.290	1.371	<.0001^a^
Age				
70–79 (standard = 60–69)	1.879	1.824	1.936	<.0001^a^
≥80 (standard = 60–69)	2.379	2.216	2.553	<.0001^a^
Urban resident (standard = rural resident)	1.329	1.293	1.366	<.0001^a^
Eligibility				
Family member (standard = head of household)	1.005	0.975	1.035	0.7450
Medical aid beneficiaries (standard = head of household)	1.499	1.416	1.588	<.0001^a^
Income level				
Second quintile (standard = first quintile)	0.979	0.930	1.032	0.4328
Third quintile (standard = first quintile)	0.947	0.900	0.996	0.0353^a^
Fourth quintile (standard = first quintile)	0.939	0.896	0.984	0.0084^a^
Fifth quintile (standard = first quintile)	0.952	0.911	0.995	0.0290^a^
History of periodontal treatment (standard = no history)	0.961	0.931	0.991	0.0113^a^
History of dental caries (standard = no history)	1.064	1.032	1.097	<.0001^a^

^a^Statistically significant at a 95% confidence interval

We examined the effects of tooth loss and specific variables on the onset of dementia by adjusting for other confounding factors. Without adjusting for confounding factors, the risk of dementia increased with age. Therefore, we analyzed the correlation between age and dementia incidence by adjusting for the effects of gender, residential area, household income, health insurance eligibility, and history of dental caries and periodontal treatment. We found that age had a statistically significant effect on the correlation between the incidence of dementia and tooth loss (*p* < 0.01). The OR for the incidence of dementia was 1.23 (95% CI: 1.186–1.276) for the 60–69 group, 1.08 (95% CI: 1.023–1.133) for the 70–79 group, and 1.32 (95% CI: 1.323–1.540) for the ≥80 group. Next, we analyzed the correlation between sex and dementia incidence by adjusting for the variables listed above. Sex also had a statistically significant effect on the correlation between the incidence of dementia and tooth loss (p < 0.01). The male and female ORs for the incidence of dementia were 1.30 (95% CI: 1.233–1.379) and 1.14 (95% CI: 1.099–1.176), respectively.

Finally, we analyzed the correlation between age and the number of teeth lost on the incidence of dementia by adjusting for the variables listed above (Table 5). The correlation between the incidence of dementia with the number of lost teeth and age was statistically significant for all age groups (*p* < 0.05). The OR tended to increase as the number of teeth lost increased for all ages, and a higher age coincided with a higher OR. The group with ≥13 teeth missing in the ≥80 group had the highest OR, although the deviation was large.

**Table 5:** Relationship between number of lost teeth and incidence of dementia by age group

Age	Tooth lost	Odds Ratio	95% Wald Confidence Limits		*p*-value
60–69	1–6	1.209	1.164	1.257	<.0001a
	7–12	1.319	1.238	1.406	<.0001^a^
	≥13	1.333	1.163	1.528	<.0001^a^
70–79	1–6	1.056	1.001	1.113	0.0457^a^
	7–12	1.165	1.068	1.271	0.0005^a^
	≥13	1.230	1.026	1.475	0.0251^a^
≥80	1–6	1.288	1.101	1.506	0.0015^a^
	7–12	1.522	1.161	1.994	0.0023^a^
	≥13	1.864	1.027	3.383	0.0405^a^

^a^Statistically significant at a 95% confidence interval

## Discussion

Individuals with tooth loss had a higher risk for dementia than those without tooth loss in this study using the NHIS ECD. Regarding the incidence of dementia, the OR increased as the number of missing teeth and age increased, and the OR was higher for women than for men, and this difference was statistically significant (*P* < 0.01). The incidence of dementia decreased with periodontal treatment and increased with dental caries.

In population studies, it is difficult to guarantee accuracy because we must indirectly infer the information using coded diagnostic and treatment information. In this study to prospectively identify the relationship between the onset of dementia and tooth loss, we would ideally determine the number of teeth remaining at the beginning of the study and the number lost during the observation period as well as identify the difference in the incidence of dementia. However, the ECD did not record the oral health status of subjects at the start, so we were unable to specify the number of remaining teeth at the time of dementia diagnosis. Therefore, we chose to investigate how many teeth were extracted during the observation period using the medical history data. Through this, we confirmed whether there were teeth lost during the study period and compared the number of extracted teeth.

In the ECD, patients with dementia were defined using disease codes. In a study of the association between dementia and tooth loss, various tests, including the Mini-Mental State Examination (MMSE), Delayed Word Recall Method, Beck’s Depression Inventory, and Verbal Fluency Performance Test, were used to define dementia [4, 5, 8–10, 13, 15–18, 31]. According to the NHIS insurance benefit standards, to access dementia treatment, patients should be evaluated by the MMSE and Clinical Dementia Rating (CDR), and dementia symptoms should meet certain score criteria (See Additional file 7) [34]. Therefore, the subjects with dementia in this study included people who fulfilled the criteria according to the MMSE and CDR tests, making the accuracy of the diagnosis somewhat objective.

We also used treatment codes to define tooth extraction. There are eight major causes of adult tooth extraction: dental caries, periodontal disease, orthodontic treatment, trauma, prosthodontic treatment, patient needs, pericoronitis of the third molar, and other undefined reasons [35]. Insurance in Korea does not cover tooth extractions for orthodontic treatment, prosthodontic treatment, patient request, or unexplained reasons, so those extractions were not included in the ECD. In this study, we included tooth extractions due to dental caries, periodontal disease, and trauma, and only had to rule out extraction of the third molar due to pericoronitis, which could be excluded based on disease code or treatment code. The extractions of impacted teeth (U4415, U4416, and U4417) associated with third molars and those of deciduous teeth (U4411) not associated with older adults were also excluded. Finally, we only included U4412 (Extraction-Anterior tooth), U4413 (Extraction-Posterior tooth) and U4414 (Extraction-Complicated Extraction).

The tooth extraction cohort was divided into three groups according to the total number of teeth lost over 10 years (1–6, 7–12, and ≥ 13 teeth lost). Many researchers have tried to classify tooth loss [6–8, 28, 32]. In this study, we grouped the number of missing teeth based on chewing efficiency. The loss of six teeth was regarded as the maximum loss that would permit chewing on one side of the mouth with posterior teeth. If 12 teeth were lost, chewing is possible with premolars or with the help of removable partial dentures. The group with ≥13 teeth lost was considered to have a significant reduction in chewing function. Because unified criteria have not been established, it will be necessary to determine grouping criteria for future effective comparative studies.

The demographic characteristics of the two cohorts classified by history of extraction showed that a smaller percentage of patients received medical aid in the extraction cohort than in the non-extraction cohort. The insurance service tried to increase the medical access of beneficiaries by supporting higher rates of medical expenses and lowering their payments [36]. However, because prosthetic treatment after tooth extraction is not covered by insurance benefits, the proportion of medical aid beneficiaries in the extraction cohort decreased. In addition, the proportion of the population with the highest income was larger in the extraction cohort than in the non-extraction cohort. It seems probable that these people were more likely to use medical facilities because their income was higher.

This study shows that the number of extracted teeth is associated with higher risk of dementia. The risk of dementia increased with the number of teeth lost. It also increased for the 60–69 and ≥ 80 age groups and was higher in males than females. These results are similar to those of previous studies. Many previous studies found a correlation between the incidence of dementia or cognitive dysfunction and increased tooth loss [4, 13, 14, 16]. Similar results were obtained in this study of more than 100,000 people. However, some studies found that the number of missing teeth is not associated with dementia or cognitive dysfunction or found a negative correlation between the two [19, 24, 31, 32]. These studies had relatively small sample sizes, and some acknowledged a partial association between tooth loss and dementia, indicating that caution should be exercised when interpreting the results.

Although previous studies have suggested a relationship between tooth loss and dementia, the order of these occurrences is unclear [4, 13, 14, 16]. Several researchers have proposed different reasons for the association between tooth loss and dementia. One is that dementia is a contributing risk factor for tooth loss, and another suggests that tooth loss is the leading factor for dementia. The view that dementia acts as a leading factor suggests that dementia and cognitive dysfunction might increase the risk of tooth loss because the disease makes managing oral hygiene, visiting dental clinics, and receiving dental treatment difficult [17, 37, 38]. There are two hypotheses about adverse causal relationships. 1) Food intake is reduced due to loss of teeth, resulting in nutritional imbalance and a lack of nutrients like vitamin B, which is essential for cognitive function [39, 40]. 2) Periodontal disease causes tooth loss and produces inflammatory response precursors [41–45]. These precursor, which are secreted as a part of the immune function, could migrate to the blood-brain barrier, causing an inflammatory response that leads to vascular pathogenesis and possibly neuronal atrophy in the brain. Tests of these hypotheses will require well-planned, randomized, controlled future studies.

Several previous studies have suggested a correlation between periodontal disease and dementia or impairment of cognitive function [4, 6, 10, 46]. Our study showed that a history of periodontal treatment was associated with increased tooth loss but also with a significantly reduced risk of dementia. This contrast with the increase in risk of dementia incidence when considering only the number of tooth extractions. Taken together, these data may indicate that proper treatment of periodontal disease might lower the incidence of dementia, supporting the importance of periodontal treatment, especially in early periodontal disease. Therefore, to prevent cognitive dysfunction associated with the periodontal disease, it may be advisable to educate dementia risk groups to thoroughly manage oral hygiene and use health policies to reduce periodontal disease through treatment at the early disease stages.

Although this work suggests that tooth loss and the number of lost teeth are related to dementia, there are some limitations. First, the reliability of results from a cohort study increases when the experimental and control groups are identical at the beginning of the study. However, this study design may bias the outcome because the oral health status of the study subjects was not equivalent at the start of the study. For example, it is known that more cognitive decline occurs in fully edentulous patients, but if the patient was already fully edentulous at the beginning of the study, there was no tooth loss during the observation period. Second, studies using the NHIS database depend on the disease codes and treatment codes recorded in the database. The database used in this study includes only diagnostic information and information on benefit treatments covered by health insurance. Disease codes listed in the cohort may not represent a participant’s true disease status because the code was created to claim health insurance services [27]. It can be difficult to trust the results of the study for codes entered that may be different from the actual diagnosis or treatment. This also makes it difficult to assess the impact of important confounding factors such as smoking and alcohol, which are not included in the NHIS-NSC database. Third, because the ECD only includes data from 2002 to 2015, longer-term observational studies are not yet available. Fourth, although the presence or absence of comorbidities would affect the onset of dementia, the effects of other systemic diseases were not considered. In addition, the differences in the incidence of dementia based on the type of extraction, dementia or periodontal therapy were not analyzed, and further studies are needed. Despite these limitations, this study is meaningful because it is a population-based study with a large number of subjects [47].

## Conclusions

We compared the risk for the onset of dementia between individuals who underwent tooth extraction and those who did not using the NHIS ECD. We found that individuals with tooth loss had a higher risk for dementia than individuals without tooth loss regardless sex, age, and the number of teeth lost. The incidence of dementia increased as the number of missing teeth increased, and it decreased when periodontal treatment was performed. From this perspective, individuals should maintain oral hygiene and healthcare policies should facilitate access to oral health care for those who have difficultly managing their own oral hygiene. Conversely, early detection of dementia may be aided by evaluating cognitive function in patients with extensive teeth loss.

## Additional files

Additional file 1: Treatment codes and definition of behaviors associated with tooth extraction. (XLSX 9 kb)

Additional file 2: Disease codes and definitions associated with dementia. (XLSX 9 kb)

Additional file 3: Disease codes and definitions associated with periodontal diseases. (XLSX 9 kb)

Additional file 4: Treatment codes and definitions of behaviors associated with periodontal diseases. (XLSX 10 kb)

Additional file 5: Disease codes and definitions associated with dental caries. (XLSX 9 kb)

Additional file 6: STROBE Statement. Checklist of items that should be included in reports of cohort studies. (DOCX 28 kb)

Additional file 7: Anti-dementia drugs and requirements for claiming NHIS insurance benefits. (XLSX 9 kb)

## Abbreviations

CDR: Clinical Dementia Rating
ECD: Elderly Cohort Database
GDS: Global Deterioration Scale
IRB: Institutional Review Board
MMSE: Mini-Mental State Examination
NHIS-ECD: National Health Insurance Service-Elderly Cohort Database
NHIS-NSC: National Health Insurance Service–National Sample Cohort
OR: Odds ratio
